# Virtual reality mindfulness-based cognitive therapy for reducing psychological distress in people with depression and/or anxiety symptoms: two uncontrolled feasibility studies

**DOI:** 10.1186/s40814-026-01892-w

**Published:** 2026-08-04

**Authors:** Rebecca L. Gould, Matthew Hoad-Robson, Kim Bevan, Amy Bedingham

**Affiliations:** 1 Division of Psychiatry, University College London, London, UK; 2 Tend VR, London, UK

**Keywords:** Virtual reality, Mindfulness-based cognitive therapy, Feasibility, Acceptability, Depression, Anxiety

## Abstract

**Background:**

Group mindfulness-based cognitive therapy (MBCT) is recommended for the treatment of subthreshold-to-mild depressive symptoms. Timely access to MBCT, however, is limited by accessibility issues. Self-help MBCT can overcome these issues, but is associated with poorer engagement. Incorporating virtual reality into self-help MBCT (VR-MBCT) may improve engagement, but relatively little is known about its feasibility and acceptability. Consequently, the aims of this study were to examine the acceptability, feasibility and preliminary signals of efficacy of VR-MBCT for reducing psychological distress in people with depression and/or anxiety symptoms.

**Methods:**

In two uncontrolled feasibility studies, participants aged ≥ 18 years with mild-to-moderately severe symptoms of depression and/or anxiety were recruited via self-referral. Participants received five VR-MBCT sessions (totalling approximately two hours in duration) each week for 8 weeks plus usual care. Co-primary outcomes were feasibility (recruitment, retention and data completion) and acceptability (course completion and satisfaction with VR-MBCT) in both studies. Secondary outcomes included measures of depression, anxiety, mental well-being and mindfulness, assessed at pre- and post-intervention.

**Results:**

In Study 1, 35 participants (100% of the target) were recruited, 22 participants (63%) were retained, 20–22 participants (57–63%) completed outcome measures at both timepoints, and 18 participants (51%) completed 4 weeks of VR-MBCT. In Study 2, 48 participants (96% of the target) were recruited, 26 participants (54%) were retained, 23–26 participants (48–54%) completed outcome measures at both timepoints, and 22 participants (46%) completed 4 weeks of VR-MBCT. Satisfaction with VR-MBCT was high in both studies. Data were suggestive of improvements in anxiety, depression, mental well-being and mindfulness from pre- to post-intervention in both studies. Rates of reliable improvement were 77–90% in Study 1 and 62–70% in Study 2.

**Conclusions:**

There was good evidence of the acceptability of VR-MBCT with respect to course satisfaction and feasibility in terms of recruitment. Although consistent with previous studies, findings suggested that further improvements could be made regarding retention/attrition, data completion and initial engagement. Preliminary signals of efficacy suggested that VR-MBCT may reduce psychological distress and improve mental wellbeing and mindfulness in people with depression and/or anxiety symptoms. However, results should be interpreted with caution due to study limitations.

**Supplementary Information:**

The online version contains supplementary material available at https://doi.org/10.1186/s40814-026-01892-w.

## Key messages regarding feasibility

- To the authors’ knowledge, no studies have evaluated the acceptability, feasibility and preliminary signals of efficacy of virtual reality mindfulness-based cognitive therapy (VR-MBCT) for reducing psychological distress (i.e., depression and anxiety) in people with depression and/or anxiety symptoms.
- Results indicated that there was good evidence of the feasibility of VR-MBCT in terms of recruitment and acceptability with respect to course satisfaction. However, findings suggested that further improvements could be made with respect to retention, attrition, data completion and initial engagement, since rates were adequate (though consistent with previous studies).
- There was evidence of preliminary signals of efficacy with respect to improvements in depression, anxiety, mental wellbeing and mindfulness. However, caution should be expressed in interpreting these results due to study limitations (including the lack of a control group and follow-up outcome assessment and potential self-selection bias).
- Overall, findings highlight that a fully powered randomised controlled trial (RCT) of VR-MBCT for reducing psychological distress in people with depression and/or anxiety symptoms is warranted. However, minimising attrition and maximising initial engagement and data completion are key issues that will need to be successfully addressed before this can occur.

## Background

Mindfulness-based cognitive therapy (MBCT) is a psychological intervention that combines mindfulness-based stress reduction with elements of cognitive behavioural therapy (CBT) [1–3]. Originally developed as an 8-week, in person, group intervention for the prevention of depressive relapse in those in remission, MBCT has since been applied to numerous other clinical populations, including those experiencing current depression and anxiety symptoms [4]. Previous systematic reviews and meta-analyses have shown that MBCT is superior to treatment-as-usual for preventing relapse in depression and delaying time to relapse [5, 6], as well as being as efficacious as CBT in the treatment of current depression [7]. In recognition of this, National Institute for Health and Care Excellence (NICE) guidelines [8] recommend MBCT for (i) the prevention of depressive relapse in adults with recurrent depression; and (ii) the treatment of current “less severe” depression (i.e., subthreshold and mild depression) in adults who do not wish to receive CBT or Behavioral Activation.

Despite these recommendations, group MBCT remains inaccessible to some people due to pragmatic and logistical issues, as well as personal preferences. These include the fact that attendance relies on people being able to fit their schedules around fixed group session times, which may not be possible for all (such as carers and shift workers). Another issue is that MBCT may not be available within local clinical services, as noted in a systematic exploration of the implementation of MBCT across the UK. This highlighted difficulties in accessing MBCT within NHS services, describing access as “patchy and variable” (p9) [9]. Personal preferences with respect to engaging in group interventions or perceived stigma in relation to mental health may also serve as a barrier to some [10]. Consequently, alternative means of accessing MBCT are required.

Guided self-help MBCT interventions represent a potentially more accessible and cost-effective solution to the issues noted above. For example, recent randomised controlled trials (RCTs) have shown that practitioner-supported, self-help MBCT is not only superior to usual care for reducing depressive symptoms in those experiencing mild to moderate symptoms of depression, but also superior to other treatments such as practitioner-supported, self-help CBT [11, 12]. However, the fact that guided self-help MBCT still requires therapeutic input means that people may not receive immediate or timely access to it, as this will depend on waiting times and availability of trained practitioners within local clinical services. While unguided self-help MBCT interventions (including online interventions and apps) may offer an alternative means of resolving this access issue, such interventions are typically subject to poor engagement, as evidenced by low adherence and high attrition rates, respectively. For example, systematic reviews have reported adherence rates ranging from 40 to 92% and mean attrition rates ranging from 31 to 39% [13–15].

One way of potentially overcoming all of the barriers noted thus far is by incorporating immersive technologies such as virtual reality (VR) technology into unguided self-help MBCT. In doing so, this may retain the advantages of accessibility and timely access that unguided self-help MBCT offers, but may also overcome engagement issues due to the immersive and stimulating nature of VR. In support of this, a recent systematic review of VR-based mindfulness training reported that it was more effective than conventional mindfulness training in improving mindfulness levels [16]. These authors suggested this may have been due to VR reducing external distractions and hence enhancing attentional focus. Another systematic review reported both psychological and physiological benefits of VR-based mindfulness interventions [17]. However, the majority of studies in both of these reviews were brief, single-session interventions and none were based on the MBCT programme. Therefore, it remains unclear as to whether unguided self-help VR-based MBCT (hereafter referred to as VR-MBCT) is a viable alternative to group MBCT, guided self-help MBCT or unguided self-help MBCT.

To the authors’ knowledge, no published studies to date have evaluated VR-MBCT for reducing psychological distress in people with depression and/or anxiety symptoms. Consequently, the primary aims of the studies reported here were to examine the feasibility and acceptability of VR-MBCT for people with depression and/or anxiety symptoms. Secondary aims were to obtain preliminary estimates of “signals of efficacy” of VR-MBCT for reducing psychological distress (i.e. depression and anxiety symptoms) and improving mental well-being and mindfulness.

## Study 1

### Materials and methods

This study is reported in accordance with the Consolidated Standards of Reporting Trials (CONSORT) guidelines [18] and the Template for Intervention Description and Replication guidelines [19]. Associated checklists are provided in Additional files 1–2. Ethical approval was granted by The Retreat Clinics’ Clinical Research Ethics Committee on 4th September 2023.

#### Design

An uncontrolled, feasibility study.

#### Participants and setting

Participants were recruited via self-referral from advertisements on Tend VR’s website (https://www.tendmentalhealth.com/) and social media (e.g., Instagram and Facebook), and advertisements on the websites and social media of voluntary sector partners, including The Retreat Clinics (York and Manchester), York Mind and Inspire North in England and New Horizons in Wales. In addition, voluntary sector partners alerted their networks to the opportunity by email.

#### Inclusion and exclusion criteria

Participants were eligible if they

1. Were aged ≥ 18 years;
2. Scored 5–19 on the Patient Health Questionnaire-9 (PHQ-9) [20] and/or scored 5–18 on the Generalised Anxiety Disorder Questionnaire-7 (GAD-7) [21];
3. Were not reporting active suicidal ideation and intent or risk of harm to self or others;
4. Had sufficient ability to understand written and spoken English to provide informed consent and to participate in the MBCT cognitive therapy programme
5. Were able to commit to completing the full MBCT programme;
6. Had access to Wi-Fi and a private space for the use of the VR headset and had sufficient familiarity with IT equipment to understand operating instructions.

Exclusion criteria included

1. Experiencing current hallucinations;
2. Physical health conditions that would interfere with the headset technology (e.g., experiences severe motion sickness, has photosensitive epilepsy or wears strong prescription glasses);
3. Currently receiving psychotherapy;
4. Life-limiting illnesses where the participant was not expected to survive the duration of the study;
5. Moderate or severe learning disability;
6. Current or imminent major life changes.

#### Intervention

Core aspects of traditional MBCT practice were selected using a combination of literature review, technical capability testing and outcomes from a previous proof-of-concept study to create a VR-based MBCT programme. This comprised five sessions totalling approximately two hours in duration each week for 8 weeks, delivered via a Meta Quest 2 headset (that was loaned for the duration of the intervention period) within the participant’s home. The five weekly sessions were distributed over 5–7 days per week, with each session lasting 12–20 min in duration, depending on the engagement of the user and the content of the session. Participants were encouraged to complete the 8-week course within 12 weeks. They were also encouraged to practise skills developed within the programme and apply them to their daily life.

Key components of the programme included formal and informal mindfulness practices and psychoeducation taken from MBCT, such as breath-based mindfulness practices, body scans and exercises focusing on awareness of automatic thinking (see Additional file 3). In addition, the programme incorporated gamification elements in order to increase the likelihood of sustained engagement and interest with it. These included a tree that grows to demonstrate user progress through the course, unlockable wildlife which populate the virtual world acting as rewards after completing sections of the course, as well as streaks and other achievements.

#### Usual care

All participants received usual care in addition to VR-MBCT. This included treatment with antidepressants and monitoring within primary care services, though currently receiving psychotherapy was an exclusion.

#### Procedure and data collection

All recruitment adverts included a link to an online expression-of-interest JotForm (which included the Participant Information Sheet) and a taster video of VR-MBCT. Potential participants were asked to read the Participant Information Sheet and, if still interested in participating in the study, complete an online expression-of-interest form (via JotForm) and an initial consent form. They were also asked to provide key socio-demographic and clinical data relating to eligibility criteria. In addition, they were asked to complete two screening measures, the PHQ-9 and GAD-7 [20, 21], as well as questions related to risk, in order to determine their eligibility for participation in the study. If they scored the maximum permitted score on the PHQ-9 or GAD-7 for the study (i.e., 19 and 18, respectively) and/or their responses to the risk questions indicated possible risk then their form was reviewed by a clinician from The Retreat Clinics. If necessary, the potential participant was invited to attend a 15-min telephone screening interview with a clinician from The Retreat Clinics to further check eligibility.

Participants who met eligibility criteria and provided fully informed consent via an online JotForm were invited to participate in the study. They were asked to complete two further pre-intervention questionnaires via an online JotForm: the Warwick–Edinburgh Mental Wellbeing Scale (WEMWS) [22] and the Southampton Mindfulness Questionnaire (SMQ) [23]. A VR headset was then delivered to each participant’s home and the participant was given a quick start guide to the VR technology and access to the VR-based MBCT programme. Participants were invited to complete post-intervention questionnaires via an online JotForm at 8 weeks, and a proportion of participants were invited to participate in a qualitative interview (data reported elsewhere).

#### Outcomes

The co-primary outcomes were (i) feasibility as defined by the number of participants who were recruited and retained and data completeness with respect to secondary outcome measures; and (ii) acceptability as defined by course completion rates, with those who completed 4 weeks or more (i.e. considered to be an adequate dose of MBCT [24]) being categorised as course completers, and satisfaction with the intervention. The latter outcome was measured using two questions: (1) “Would you recommend MBCT to your friends and family?” which was rated on a 5-point Likert scale from 1 (would not recommend) to 5 (very strongly recommend); and (2) “How would you describe your experience of VR-MBCT?” which was rated on a 5-point Likert scale from 1 (very negative experience) to 5 (very positive experience). Secondary outcomes included additional measures of acceptability and feasibility: (i) attrition due to lack of acceptability of the intervention and attrition for feasibility reasons; and (ii) reasons for course non-completion in relation to acceptability and feasibility.

Secondary patient-reported outcomes included the following measures, which were assessed pre- and post-intervention:

i) PHQ-9 [20]: a 9-item measure of depression, which is commonly utilised in primary care settings;
ii) GAD-7 [21]: a 7-item measure of generalised anxiety disorder, which is also commonly used in primary care settings
iii) Warwick–Edinburgh Mental Wellbeing Scale (WEMWS) [22]: a 14-item measure of mental well-being;
iv) Southampton Mindfulness Questionnaire (SMQ) [23]: a 16-item process measure of mindful responses to thoughts and images.

#### Data analyses

Continuous measures were summarised using means and standard deviations (SDs) and categorical measures were summarised using frequencies and percentages. As recommended in pilot and feasibility studies [25, 26], no formal statistical data analysis was conducted and so changes in outcomes were reported descriptively. Change scores across time were calculated for participants who had completed patient-reported outcome measures at both pre- and post-intervention, and then averaged across participants. As recommended for paired data [27], Cohen’s *d* effect sizes (with confidence intervals) were calculated by dividing the mean change score by the SD of the change scores. Reliable Change Index (RCI) and Clinically Significant Change (CSC) scores were calculated using the Leeds Reliable Change Indicator calculator [28] to determine whether change scores across time were reliable (i.e. not explained by repeated measurement artefacts or measurement error). RCI calculations were based on published estimates of internal consistency.

#### Sample size

A sample size of 35 participants was chosen following conventional recommendations for sample sizes ranging from 24 to 35 for pilot and feasibility studies [29–31]. This accounted for an estimated attrition rate of 31% at post-intervention based on a mean attrition rate of 31% reported in a systematic review of unguided mindfulness-based self-help interventions [15].

### Results

#### Study flow

**Fig. 1:**
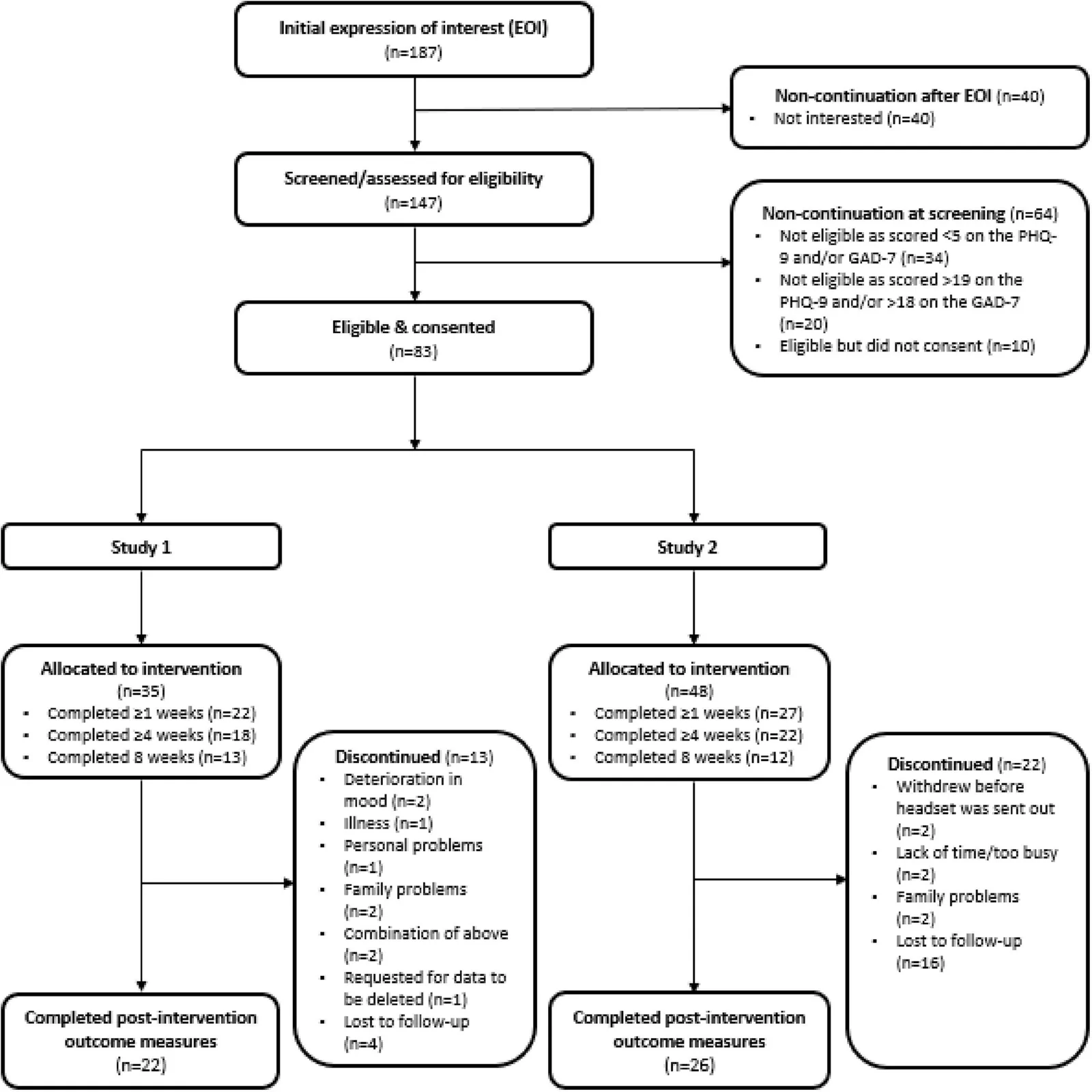
Summary of recruitment and follow-up of participants

As shown in Fig. 1, initial expressions of interest were received from 187 potential participants between 21 August 2023 and 29 September 2023, 79% (147/187) of whom were assessed for eligibility. Of these, 56% (83/147) were eligible and consented to participate, with 35 participants recruited to Study 1 and 48 participants recruited to Study 2 (described later). There was a 37% dropout rate (13/35), with four participants being lost to follow-up.

#### Baseline characteristics

Baseline demographic and clinical characteristics are described in Table 1. The majority of participants self-identified as female (24/35, 71%), White (31/35, 91%) and employed (29/35, 83%), with a mean age of 43.5 years (SD 12.4). Three quarters of participants self-reported a mental health diagnosis (26/35, 74%), with mixed anxiety and depression being most commonly reported (16/26, 62%). Just under half reported being prescribed psychotropic medication (16/35, 46%), but only a small proportion reported involvement with mental health services (5/35, 14%).

**Table 1:** Demographic and clinical characteristics of participants at baseline

	Study 1 (*N* = 35)		Study 2 (*N* = 48)	
Variable	*N* (missing *N*, %)	Mean (SD) or *N* (%)	*N* (missing *N*, %)	Mean (SD) or *N* (%)
Mean age (years)	34 (1, 3%)	43.5 (12.4), range 20–76	45 (3, 6%)	45.2 (10.4), range 24–69
Age category (years)	34 (1, 3%)		45 (3, 6%)	
≤ 64		33 (97%)		44 (98%)
≥ 65		1 (3%)		1 (2%)
Gender	34 (1, 3%)		45 (3, 6%)	
Female		24 (71%)		31 (69%)
Male		9 (26%)		12 (27%)
Other		1 (3%)		2 (4%)
Ethnicity	34 (1, 3%)		45 (3, 6%)	
White		31 (91%)		41 (91%)
Other		3 (9%)		4 (9%)
Employment status	34 (1, 3%)		45 (3, 6%)	
Paid or self-employed		29 (83%)		43 (90%)
Unemployed		2 (6%)		1 2%)
Retired		1 (3%)		1 (2%)
Other		2 (6%)		
Self-reported mental health diagnosis	34 (1, 3%)		45 (3, 6%)	
Yes		26 (74%)		29 (60%)
No		8 (23%)		16 (33%)
Type of self-reported mental health diagnosis	26 (0, 0%)		28 (1, 3%)	
Anxiety		5 (19%)		6 (21%)
Depression		4 (15%)		5 (17%)
Anxiety and depression		16 (62%)		17 (59%)
Other		1 (4%)		0 (0%)
Prescribed psychotropic medication	34 (1, 3%)		45 (3, 6%)	
Yes		16 (46%)		14 (29%)
No		18 (51%)		31 (65%)
Involvement with mental health services	34 (1, 3%)		45 (3, 6%)	
Yes		5 (14%)		1 (2%)
No		29 (83%)		44 (92%)

*SD* standard deviation

#### Intervention completion

**Fig. 2:**
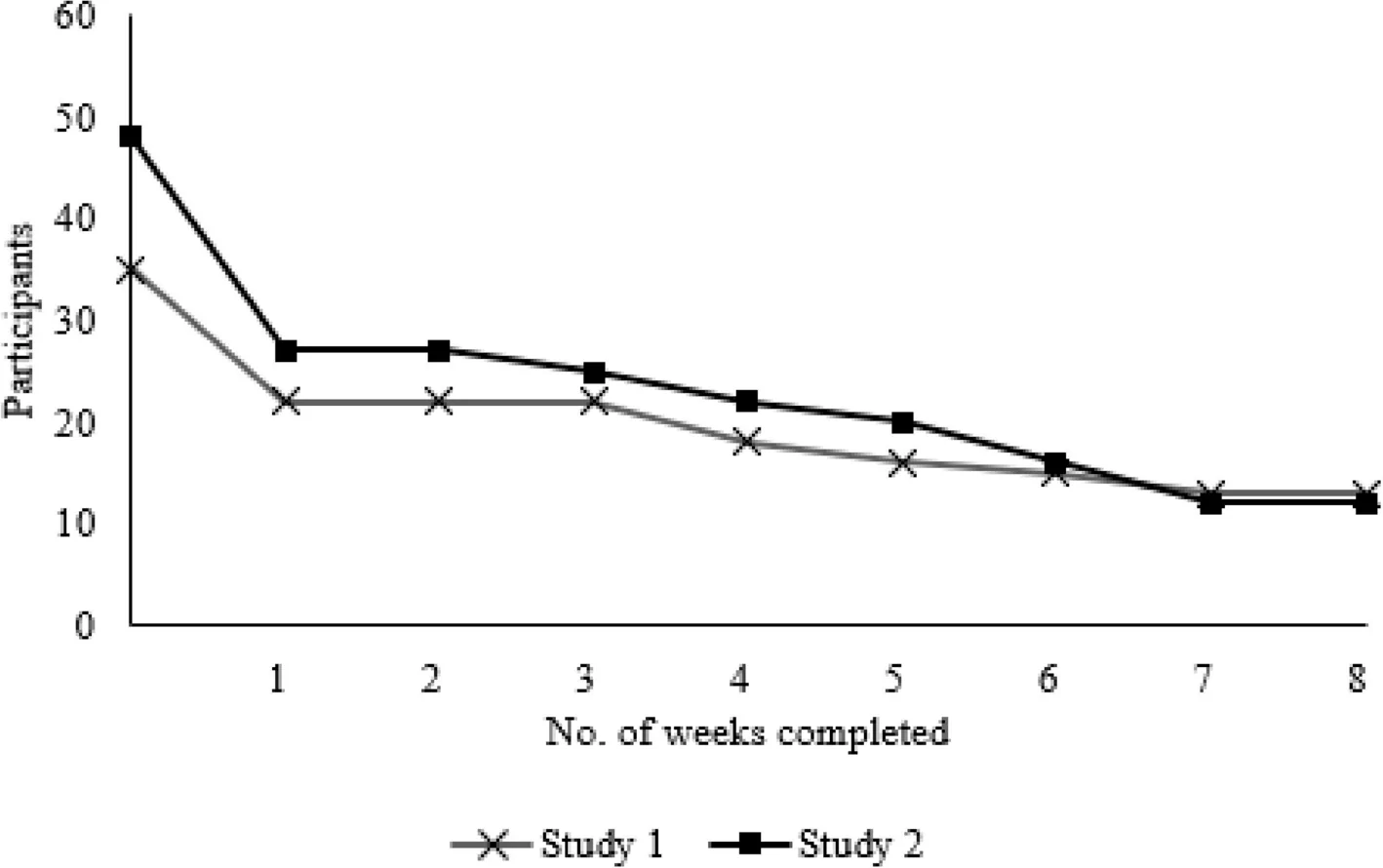
Intervention completion per week

The median number of weeks of the intervention completed was 8.0 (interquartile range [IQR] 4.0), with 63% of participants (22/35) completing the first week, 51% (18/35) completing 4 weeks and 37% (13/35) completing all 8 weeks (see Fig. 2). The majority of participants who completed the first week proceeded to complete 4 weeks (82%, 18/22), with 59% of these (13/22) completing all 8 weeks.

#### Primary outcomes

Results for the co-primary outcomes with respect to feasibility and acceptability are shown in Table 2.

**Table 2:** Co-primary outcomes with respect to feasibility and acceptability in Studies 1 and 2

	Study 1 (*N* = 35)		Study 2 (*N* = 48)	
Outcome	*N* (%)	Mean (SD)	N (%)	Mean (SD)
No. of participants recruited (% of target sample)	35 (100%)	–	48 (96%)	–
No. of participants retained	22 (63%)	–	26 (54%)	–
No. of participants who dropped out	13 (37%)	–	22 (46%)	–
No. of participants completing 4 weeks	18 (51%)	–	22 (46%)	–
Satisfaction with VR-MBCT				
Recommend to friends and family^a^	22	4.36 (0.79)	26	4.19 (0.98)
Positive experience^b^	22	4.27 (0.88)	26	4.15 (1.01)

^a^Rated on a scale from 1 (would not recommend) to 5 (very strongly recommend)

^b^Rated on a scale from 1 (very negative experience) to 5 (very positive experience)

##### Feasibility

One hundred percent of the target sample (35/35) was recruited within the two-month recruitment period. Sixty-three percent (22/35) of participants were retained in the study at post-intervention. Of the participants who withdrew from the study, 62% (8/13) did so due to feasibility issues that were unrelated to the intervention (e.g., physical or mental health, personal or social issues). Outcome measures were completed at both timepoints by 57–63% (20/35–22/35) of participants (see Additional File 4). Reasons for course non-completion were unknown in the majority of participants (13/17, 76%). In the few cases where reasons were provided, three (18%) were due to feasibility issues (lack of time and social issues).

##### Acceptability

Fifty-one percent of participants (18/35) completed 4 weeks of the intervention, and so were categorised as completing the course. No participants withdrew from the study due to reported lack of acceptability of the intervention. Course non-completion due to an acceptability issue with the equipment (motion sickness from headset) occurred in one out of 17 participants (6%). However, of the withdrawals, four participants were lost to follow-up and one participant requested for their data to be deleted (5/13, 38%) and therefore it is unclear whether lack of acceptability was a contributing factor in these cases. Mean satisfaction ratings in those completing post-intervention measures were high (see Table 2). Of those who provided ratings (22/35), 82% would strongly or very strongly recommend VR-MBCT to friends and family, and 77% rated themselves as having a positive or very positive experience of VR-MBCT.

#### Secondary outcomes

Data suggested decreases in depression and anxiety symptoms, in addition to increases in mental wellbeing coupled with increases in mindfulness, from pre- to post-intervention (see Table 3). There was a 7-point reduction between pre-intervention and post-intervention for both depression (PHQ-9 7.46; 95% CI 5.24 to 9.67) and anxiety (GAD-7 7.64; 95% CI 5.61 to 9.66). There was also an 11-point increase in mental wellbeing (WEMWBS − 11.85; 95% CI − 15.05 to − 8.65) and a 21-point increase in mindfulness (SMQ − 21.95; − 29.85 to − 14.05). Cohen’s *d* pre–post effect sizes were large in magnitude, ranging from 1.30 for mindfulness to 1.74 for mental wellbeing.

**Table 3:** Mean scores at pre- and post-intervention, mean change scores and effect sizes

Secondary outcome	*N*	Pre Mean (SD)	*N*	Post Mean (SD)	Mean change score (pre–post) Estimated change (95% CI, SD)	Cohen’s *d* pre–post effect size (95% CI)
Study 1 (*N* = 35)						
PHQ-9	34	13.97 (3.42)	22	6.32 (4.50)	7.46 (5.24 to 9.67, 4.99)	1.49 (0.87 to 2.10)
GAD-7	34	11.71 (4.06)	22	5.50 (3.52)	7.64 (5.61 to 9.66, 4.57)	1.67 (1.01 to 2.32)
WEMWBS	33	37.39 (7.74)	20	47.30 (6.38)	− 11.85 (− 15.05 to − 8.65, 6.83)	− 1.74 (− 2.43 to − 1.02)
SMQ	33	32.27 (11.62)	20	48.90 (13.45)	− 21.95 (− 29.85 to − 14.05, 16.87)	− 1.30 (− 1.89 to − 0.69)
Study 2 (*N* = 48)						
PHQ-9	45	9.76 (3.69)	26	5.92 (4.49)	4.23 (2.46 to 6.01,4.39)	0.96 (0.49 to 1.42)
GAD-7	45	10.02 (3.71)	26	5.50 (3.90)	4.39 (2.59 to 6.18, 4.44)	0.99 (0.51 to 1.45)
WEMWBS	40	38.05 (7.66)	23	48.57 (7.01)	− 9.00 (− 12.07 to − 5.93, 7.10)	− 1.27 (− 1.81 to − 0.71)
SMQ	40	32.95 (11.90)	23	55.04 (13.33)	− 21.91 (− 30.31 to − 13.52, 19.41)	− 1.13 (− 1.65 to − 0.60)

*CI* confidence interval, *ES* effect size, *GAD-7* Generalised Anxiety Disorder-7: possible scoring range 0–21; higher scores indicate greater anxiety, *PHQ-9* Patient Health Questionnaire-9: possible scoring range 0–27; higher scores indicate greater depression, *SD* standard deviation, *SMQ* Southampton Mindfulness Questionnaire: possible scoring range 0–96; higher scores indicate greater mindfulness, *WEMWBS* Warwick-Edinburgh Mental Wellbeing Scale: possible scoring range 14–70; higher scores indicate greater mental wellbeing

The number of participants who demonstrated reliable improvement/deterioration or no reliable change on outcome measures at post-intervention (in comparison to pre-intervention) are shown in Table 4. Reliable improvements in depression and anxiety were observed in 77% (17/22) and 86% (19/22) of participants with data at both pre- and post-intervention, respectively. Similar rates were found for mental wellbeing (90%, 18/20) and mindfulness (80%, 16/20). Clinically significant improvement rates ranged from 27% (6/22) for depression to 75% (15/20) for mental wellbeing. Importantly, no participants showed reliable deterioration on any outcome measure.

**Table 4:** Reliable change from pre- to post-intervention for those completing outcome measures at both pre- and post-intervention

Outcome measure	*N*	Cronbach’s alpha (study reference no.)	Cut-off score (study reference no.): 44 [32]	Reliable deterioration *N* (%)	No reliable change *N *(%)	Reliable improvement *N* (%)	Clinically significant improvement *N* (%)
Study 1							
PHQ-9	22	0.89 (20)	5 (20)	0 (0%)	5 (23%)	17 (77%)	6 (27%)
GAD-7	22	0.92 (21)	5 (21)	0 (0%)	3 (14%)	19 (86%)	9 (41%)
WEMWBS	20	0.91 (22)	44 (37)	0 (0%)	2 (10%)	18 (90%)	15 (75%)
SMQ	20	0.89 (23)	N/A^*^	0 (0%)	4 (20%)	16 (80%)	N/A^*^
Study 2							
PHQ-9	26	0.89 (20)	5 (20)	2 (8%)	8 (31%)	16 (62%)	11 (42%)
GAD-7	26	0.92 (21)	5 (21)	2 (8%)	6 (23%)	18 (69%)	9 (35%)
WEMWBS	23	0.91 (22)	44 (37)	0 (0%)	8 (35%)	15 (65%)	11 (48%)
SMQ	23	0.89 (23)	N/A^*^	0 (0%)	7 (30%)	16 (70%)	N/A^*^

Clinically significant improvement = improvement from pre-treatment that meets both RCI and CSC criteria (i.e. a reliable change that is also clinically significant). Reliable improvement = improvement from pre-treatment that meets RCI, but not CSC criteria (i.e. a reliable change that is not clinically significant). No reliable change = the magnitude of any change following treatment is within the expected range due to measurement error. Reliable deterioration = deterioration from pre-treatment that meets RCI, but not CSC criteria (i.e. a reliable change that is not clinically significant). Clinically significant deterioration = deterioration from pre-treatment that meets both RCI and CSC criteria (i.e. a reliable change that is also clinically significant)

*CI* confidence interval, *GAD-7* Generalized Anxiety Disorder-7: possible scoring range 0–21; higher scores indicate greater anxiety, *PHQ-9* Patient Health Questionnaire-9: possible scoring range 0–27; higher scores indicate greater depression, *SD* standard deviation, *SMQ* Southampton Mindfulness Questionnaire: possible scoring range 0–96; higher scores indicate greater mindfulness, *WEMWBS* Warwick-Edinburgh Mental Wellbeing Scale: possible scoring range 14–70; higher scores indicate greater mental wellbeing, *RCI* Reliable Change Index, *CSC* Clinically Significant Change

^*^No score available

Case levels of anxiety and depression at pre- and post-intervention in those completing outcome measures at both timepoints are shown in Table 5. The majority of participants scored in the moderately severe range for depression (13/22) and the moderate–severe range for anxiety (9/22 and 8/22, respectively) at pre-intervention. At post-intervention, most participants were scoring in the mild range for depression (11/22) and the minimal-to-mild range for anxiety (9/22 and 11/22, respectively).

**Table 5:** Case levels of anxiety and depression at pre- and post-intervention in those completing outcome measures at both timepoints

	Study 1		Study 2	
Variable (outcome measure)	Pre *N*	Post *N*	Pre *N*	Post *N*
Depression (PHQ-9)				
Severe (≥ 20)	0	0	0	0
Moderately severe (15–19)	13	1	6	2
Moderate (score 10–14)	7	3	4	2
Mild (score 5–9)	2	11	16	9
Minimal (score ≤ 4)	0	7	0	13
Missing data	13	13	22	22
Anxiety (GAD-7)				
Severe (≥ 15)	8	1	2	1
Moderate (score 10–14)	9	1	12	2
Mild (score 5–9)	5	11	11	13
Minimal (score ≤ 4)	0	9	1	10
Missing data	13	13	22	22

*GAD-7* Generalized Anxiety Disorder-7: possible scoring range 0–21, higher scores indicate greater anxiety, *PHQ-9* Patient Health Questionnaire-9: possible scoring range 0–27, higher scores indicate greater depression

##### Adverse events

There were two reports of adverse events, both of which were deemed to be related to the equipment: one participant reported motion sickness from the headset and one reported dizziness due to walking around with the headset on (contrary to instructions given). There were no reports of serious adverse events.

### Discussion

In summary, good feasibility was found in terms of recruitment, with 100% of the target sample being recruited within the recruitment window. Rates of retention (63%), attrition (37%), and outcome measure completion (57–63%) were adequate, based on overall means reported in previous systematic reviews. For example, a systematic review of unguided mindfulness-based self-help interventions reported a mean attrition rate of 31%, ranging from 0 to 79% [15]. Another systematic review of mindfulness and acceptance-based self-help interventions reported a mean outcome measure completion rate of 73%, ranging from 48 to 98% [33].

Good acceptability was observed in terms of satisfaction with the intervention, with the majority of those providing ratings recommending VR-MBCT and rating their experience of it positively. The intervention completion rate (51%) was comparable to an overall rate of 48% reported in a systematic review of unguided mindfulness-based self-help interventions [15]. The fact that the intervention completion rate rose to 82% in those who completed the first week of VR-MBCT highlights how crucial initial engagement was to intervention completion. With respect to safety, few adverse events in relation to the intervention were reported, with none related to MBCT and none classified as serious.

There was evidence of preliminary signals of efficacy with respect to improvements in depression, anxiety, mental well-being and mindfulness from pre- to post-intervention, with large Cohen’s *d* pre–post effect sizes being found for all outcome measures, ranging from − 1.30 (− 1.89 to − 0.69) to − 1.74 (− 2.43 to − 1.02). Furthermore, reliable improvements in scores were found in 77–90% of participants who completed outcome measures at both pre- and post-intervention. Importantly, these improvements were found to be clinically significant in 27–75% of these participants. These results are consistent with findings reported in previous systematic reviews of VR-based mindfulness interventions [16, 17].

In order to determine whether these promising results were replicable in a separate sample of participants, a second study evaluating the acceptability and feasibility of VR-MBCT to people with depression and/or anxiety symptoms was conducted. This second study was the same as Study 1, but included a focus on recruiting participants from an additional source located in a low socio-economic region.

## Study 2

### Materials and methods

The methodology was the same as for Study 1, with the exception of the following components:

#### Participants

In addition to recruitment sources used in Study 1, participants were also recruited from a third-sector service (New Horizons Mental Health, Welsh Valleys, Wales) in Study 2.

#### Sample size

A sample size of 50 participants was chosen, based on conventional sample size recommendations of 24–35 for pilot and feasibility studies [29–31] and accounting for an estimated attrition rate of 31% at post-intervention [15].

### Results

#### Study flow

As noted above, 48 participants were recruited for Study 2 (see Fig. 1). There was a 46% dropout rate (22/48), with 16 participants being lost to follow-up.

#### Baseline characteristics

Baseline demographic and clinical characteristics are described in Table 1. The majority of participants self-identified as female (31/48, 69%), White (41/48, 91%) and employed (43/48, 90%), with a mean age of 45.2 years (SD 10.4). Just under two thirds of participants self-reported a mental health diagnosis (29/48, 60%), with a combination of anxiety and depression being most commonly reported (17/29, 59%). Just under a third reported being prescribed psychotropic medication (14/48, 29%), but the vast majority reported no involvement with mental health services (44/48, 92%).

#### Intervention completion

The median number of weeks of the intervention completed was 6.0 (IQR 4.0), with 56% (27/48) completing the first week, 46% (22/48) completing 4 weeks and 25% (12/48) completing all 8 weeks (see Fig. 2). The majority of participants who completed the first week proceeded to complete 4 weeks (81%, 22/27), with 44% of these (12/27) completing all 8 weeks.

#### Primary outcomes

Results for the co-primary outcomes in relation to feasibility and acceptability are displayed in Table 2.

##### Feasibility

Ninety-six percent of the target sample (48/50) was recruited within the two-month recruitment period. Fifty-four percent (26/48) of participants were retained in the study at post-intervention. Of the participants who withdrew from the study, 18% (4/22) did so due to feasibility issues that were unrelated to the intervention (e.g. lack of time, social issues) A further two participants (9%) withdrew before the headset was sent out for unknown reasons. Reasons for course non-completion were also unknown in the majority of participants (19/26, 73%). In the few cases where reasons were provided, four (15%) were due to feasibility issues (withdrew before headset was sent out, personal issue, combination of feasibility issues). Outcome measures were completed at both time points by 48–54% (23/48–26/48) of participants (see Additional file 4).

##### Acceptability

Forty-six percent of participants (22/48) completed 4 weeks of the intervention, and so were categorised as completing the course. Course non-completion was due to an acceptability issue with equipment (“did not get on with the headset”, “found the headset heavy and uncomfortable”, “headset straps were difficult to adjust to fit”, and “didn’t like the feel of the headset around my face”) in 12% of participants (3/26). No participants withdrew from the study due to reported lack of acceptability of the intervention. However, of the withdrawals, 73% (16/22) did not respond when followed up and so it is unclear whether acceptability contributed to this. Mean satisfaction ratings in those completing post-intervention measures were high (see Table 2). Of those who provided ratings (26/48), 82% would strongly or very strongly recommend VR-MBCT to friends and family, and 77% rated themselves as having a positive or very positive experience of VR-MBCT.

#### Secondary outcomes

Similar to Study 1, decreases in depression and anxiety symptoms, as well as increases in mental wellbeing and mindfulness, were observed from pre- to post-intervention (see Table 3). There was a 4-point reduction between pre-intervention and post-intervention for both depression (PHQ-9 4.23; 95% CI 2.46 to 6.01) and anxiety (GAD-7 4.39; 95% CI 2.59 to 6.18). There was also a 9-point increase in mental wellbeing (WEMWBS − 9.00; 95% CI − 12.07 to − 5.93) and a 21-point increase in mindfulness (SMQ − 21.91; − 30.31 to − 13.52). Cohen’s *d* pre–post effect sizes were large in magnitude, ranging from 0.96 for depression to 1.27 for mental wellbeing.

Rates of reliable improvement in depression, anxiety, mental wellbeing and mindfulness in participants with data at both pre- and post-intervention ranged from 62% (16/26) for depression to 70% (16/23) for mindfulness (see Table 4). Clinically significant improvement rates ranged from 35% (9/26) for anxiety to 48% (11/23) for mental wellbeing. Only two out of 26 participants showed reliable deterioration in depression and anxiety (8%), and none showed reliable deterioration in mental wellbeing or mindfulness. As shown in Table 5, at pre-intervention the majority of participants scored in the mild range for depression (16/26) and the mild to moderate range for anxiety (11/26 and 12/26, respectively). Most participants were scoring in the minimal range for depression (13/26) and the mild range for anxiety (13/26) at post-intervention.

##### Adverse events

There were three reports of adverse events, all of which were related to the equipment: these participants reported experiencing discomfort due to the headset, specifically with regard to it feeling heavy and difficulties in adjusting the fit. There were no reports of serious adverse events.

### Discussion

Similar results were found in Study 2 as for Study 1, indicating that initial promising results were replicable in a separate sample of participants. Again, good feasibility was found in terms of recruitment, with 96% of the target sample being recruited within the recruitment window. Similar to Study 1, rates of retention (54%), attrition (46%), and outcome measure completion (48–54%) were adequate, based on overall means reported in previous systematic reviews [15, 33]. Satisfaction with the intervention was high, with the majority of those providing ratings recommending VR-MBCT and rating their experience of it positively, again indicating good acceptability. The intervention completion rate (46%) was comparable to an overall rate of 48% reported in a systematic review of unguided mindfulness-based self-help interventions [15]. Again, initial engagement was crucial to intervention completion, with the rate rising to 81% in those who completed the first week of VR-MBCT. No adverse events were deemed to be related to MBCT and none were classified as serious. Again, there was evidence of preliminary signals of efficacy, with large Cohen’s *d* pre–post effect sizes ranging from 0.96 (0.49 to 1.42) to − 1.27 (− 1.81 to − 0.71) being found for depression, anxiety, mental wellbeing and mindfulness. Reliable improvements in scores were found in 62–70% of participants who completed outcome measures at both pre- and post-intervention, with 35–48% of these participants showing clinically significant improvement.

### General discussion

Overall, a consistent pattern of results was found across both studies. Good feasibility was found in terms of recruitment. In contrast, retention (54–63%), attrition (37–46%), and outcome measure completion rates (48–63%) were adequate, based on overall means reported in previous systematic reviews [15, 33]. Good acceptability was observed in terms of satisfaction with the intervention. The intervention completion rate (46–51%) was comparable to a previously reported overall rate for unguided mindfulness-based self-help interventions [15]. Although the studies were not powered to examine clinical effectiveness, both studies showed preliminary signals of efficacy with respect to pre-post improvements in depression, anxiety, mental wellbeing and mindfulness, with Cohen’s *d* pre–post effect sizes being large in magnitude for all outcome measures. Moreover, improvements in scores that were both reliable and clinically significant were found for a substantial number of participants who completed outcome measures at both pre- and post-intervention in both studies.

Evidence of preliminary signals of efficacy with respect to pre–post improvements in depression, anxiety, mental wellbeing and mindfulness is consistent with previous studies. For example, improvements in numerous psychological distress and mental wellbeing outcomes, including those examined here, were reported in a systematic review of VR-based mindfulness interventions [16, 17]. As noted by the authors of these reviews, most previous studies have focused on either a single session intervention or multiple sessions of a brief intervention and none have examined MBCT. Consequently, the current findings extend previous findings in showing preliminary evidence that beneficial effects on psychological distress and mental wellbeing may also be found with VR-MBCT. Future research should examine the effectiveness of VR-MBCT in comparison to regular MBCT, as well as exploring the mechanisms by which VR may facilitate the development of mindfulness skills (such as by reducing external distractions and enhancing attentional focus [16]).

Intervention completion rates reported here are comparable to the overall rate reported in a previous systematic review of unguided mindfulness-based self-help interventions [15]. The fact that intervention completion rates rose to 81–82% in those who completed the first week of VR-MBCT (*n* = 22/35 and *n *= 27/48) highlights the importance of initial engagement. It suggests that the intervention would benefit from further refinement in order to improve initial engagement. For example, by incorporating motivational strategies, providing minimal support from clinicians or addressing issues associated with VR headsets to aid initial engagement with unguided self-help VR-MBCT. Future studies should seek to qualitatively explore barriers to and facilitators of engagement with VR-MBCT using models such as the Capability, Opportunity and Motivation model of behaviour change [34] in order to provide further suggestions for improvement.

No formal statistical analyses were conducted, as previously recommended for feasibility studies, and so comparisons across the two studies can only be descriptive in nature. An observation worth commenting on is in relation to the attrition rate, which was descriptively higher in Study 2 (46%) compared to Study 1 (37%). It is unclear why this might be the case, and it is important not to over-interpret such descriptive differences. However, one possible reason may be due to the severity of depressive symptoms at baseline, since the mean PHQ-9 score was descriptively lower in Study 2 (9.76 [SD 3.69]), corresponding to mild depression, than Study 1 (13.97 [SD 3.42]), corresponding to moderate depression. Consistent with this, self-reported psychotropic medication use was also descriptively lower in Study 2 (29%) compared to Study 1 (46%). Milder depressive symptoms and/or lower use of antidepressants at baseline may have reduced motivation or the perceived need to engage in an unguided, self-help mindfulness-based intervention. In support of this, lack of use of antidepressants has been shown to influence attrition from MBCT in a previous study [24].

The current findings with respect to retention, attrition and data completeness also suggest that adopting evidence-based procedures for retaining participants within clinical trials and encouraging them to complete outcome measures at follow-up would be beneficial in any future RCT of VR-MBCT. This might include strategies such as providing non-contingent vouchers for completion of follow-up outcome measures, building rapport with participants, sending greetings cards, personalising letters, maintaining contact through study newsletters and using study branding [35–37]. In support of this, a meta-analysis of attrition and engagement rates in RCTs of mindfulness apps reported significantly lower attrition rates in studies offering financial compensation for outcome measure completion compared to those that did not [13]. This same meta-analysis showed that studies utilising participant enrolment with no researcher contact experienced significantly higher attrition rates than those that required direct contact (either in person or via telephone or video call).

To our knowledge, no studies have explored the acceptability, feasibility, and preliminary estimates of signals of efficacy of VR-MBCT for reducing psychological distress in people with depression and/or anxiety symptoms. The studies presented here provide early evidence of the potential that VR-MBCT may offer with respect to reducing depression and anxiety symptoms and improving mental well-being in this population. However, there were limitations in the current studies. The majority of participants self-identified as White, female, employed and of working age, and so results cannot be generalised to a broader population with depression and/or anxiety symptoms. Additionally, participants were mainly recruited via self-referral, meaning that results were subject to a potential self-selection bias, further restricting the generalisability of results. The methodological design employed meant that a control group was not included, which means that it is not possible to determine whether beneficial gains were entirely due to VR-MBCT or to other factors such as expectation effects. Furthermore, participants who were currently meditating or had previously completed mindfulness courses were not excluded in order to maximise recruitment. The fact that 42% of participants (35/83) in total dropped out of Studies 1 and 2 may have resulted in an overestimation of effect sizes due to attrition bias. Therefore, results pertaining to preliminary signals of efficacy should be interpreted with caution.

With respect to recommendations for future research, the lack of follow-up outcome assessment means that it is not possible to determine whether beneficial gains were maintained beyond post-intervention. Future research should seek to evaluate VR-MBCT in comparison to an active or non-active control group, in a more diverse sample, with multiple follow-up timepoints (e.g., 3-, 6- and 12 months). Additionally, as one of the inclusion criteria was having access to Wi-Fi and a private space for the use of the VR headset and having sufficient familiarity with IT equipment to understand operating instructions, this may have excluded people from low socio-economic backgrounds or those with low digital literacy. Future research should examine whether VR-MBCT can be delivered within community settings, where Wi-Fi can be accessed and IT support can be provided, as necessary, in order to increase accessibility of the intervention. Finally, formal statistical analyses were not conducted, as recommended for pilot and feasibility studies, and so it was not possible to determine whether changes in scores for depression, anxiety and mental well-being were mediated by changes in mindfulness. Although changes in mindfulness in the current study were consistent with previous findings [33], future studies should also examine whether mindfulness mediates treatment response to VR-MBCT.

## Conclusions

Overall, there was good evidence of the feasibility of VR-MBCT in terms of recruitment and acceptability in terms of course satisfaction, in addition to evidence of preliminary signals of efficacy. However, these results should be interpreted with caution due to study limitations, including the lack of control group (given the study design), potential self-selection bias (due to the use of self-referral) and lack of follow-up assessment (as outcomes were not collected beyond post-intervention). Although the retention, attrition, engagement and data completion rates were adequate, since they were on par with previous studies of mindfulness-based self-help interventions, results suggested that further improvements should be made in these areas. If successfully addressed, findings with respect to preliminary signals of efficacy suggest that a fully powered RCT evaluating the clinical and cost-effectiveness of VR-MBCT for reducing psychological distress in people with depression and/or anxiety symptoms would be warranted.

## Supplementary Information

Additional file 1. The Consolidated Standards of Reporting Trials (CONSORT) checklist (extension for randomised pilot or feasibility trials). This file presents the CONSORT checklist for Studies 1 and 2.

Additional file 2. The template for intervention description and replication (TIDieR) checklist. This file presents the TIDieR checklist for Study 1 and 2.

Additional file 3. An outline of the VR-based MBCT weekly sessions.

Additional file 4. Data completeness for secondary outcome measures. This file provides information about data completeness for secondary outcome measures in Study 1 and 2.

## Data Availability

The datasets used and/or analysed during the current study are available from the corresponding author upon reasonable request.

## Abbreviations

CONSORT: Consolidated Standards of Reporting Trials
CSC: Clinically significant change
GAD-7: Generalised Anxiety Disorder-7
IQR: Interquartile range
MBCT: Mindfulness-Based Cognitive Therapy
NIHR: National Institute for Health and Care Research
PHQ-9: Patient Health Questionnaire-9
RCI: Reliable change index
RCT: Randomised controlled trial
SD: Standard deviation
TIDieR: Template for Intervention Description and Replication
VR: Virtual reality
