# Supplementary material for: Virtual reality mindfulness-based cognitive therapy for reducing psychological distress in people with depression and/or anxiety symptoms: two uncontrolled feasibility studies
Source: Pilot Feasibility Stud. 2026 Aug 4;12:154. doi: 10.1186/s40814-026-01892-w (PMC13721488; doi:10.1186/s40814-026-01892-w)
Supplement: Supplementary file 3 [file 40814_2026_1892_MOESM3_ESM.docx]

Additional File 3. An outline of the VR-based MBCT weekly sessions.

| **Week** | **Session** | **Brief outline of each session** |
| --- | --- | --- |
| 1 | 1 | Familiarises participants with a tool known as the "breathing ball" and explores the physiological and psychological aspects of low mood and anxiety, subsequently introducing mindfulness as a therapeutic strategy. |
|  | 2 | Elaborates on the role of breathing exercises and delves into the concept of deeply entrenched mental habits. |
|  | 3 | Participants explore how to apply breathing exercises in real-world settings beyond virtual reality, and scrutinise the notion of 'autopilot' thinking while emphasising mindfulness as an alternative. |
|  | 4 | Encourages participants to engage in introspection regarding their initial experiences and introduces a 'noticing exercise,' featuring a sunrise visualisation aimed at enhancing focus. |
|  | 5 | Commences with a grounding breath exercise and focuses on the importance and application of body scans. |
| 2 | 1 | Aims to heighten moment-by-moment bodily awareness and examines the correlation between repetitive thinking, or rumination, and low mood or depression. |
|  | 2 | Involves further practice of the body scan, followed by an investigation into automatic thinking through a thought-noticing exercise, helping people understand their cognitive shortcuts. |
|  | 3 | Continues to deepen understanding of body scans and further explores the link between automatic thinking and memories, along with common daily thoughts and emotions. |
|  | 4 | Expands the body scan technique and introduces a mindfulness exercise to reframe perceptions, emphasising the significance of understanding how emotions alter our experiences. |
|  | 5 | Involves further practice of the body scan and revisits the concept of automatic thinking, examining its role in shaping our reactions to events, locations, and emotions, capped by a reflection on instances of being in 'automatic thinking mode'. |
| 3 | 1 | Commences with a body scan, followed by an exercise focused on the perception of time and the mind's response to periods of silence. |
|  | 2 | Extends the practice of body scans and delves into one of the foundational aspects of mindfulness: awareness of automatic processes, such as breathing, complemented by related exercises. |
|  | 3 | Continues to deepen familiarity with body scans and introduces mindful movement, specifically, a seated stretching exercise. |
|  | 4 | Aims to integrate breath awareness with body scan techniques, exploring the synergy of combining these two practices. |
|  | 5 | Navigates the utility of breath in focusing the mind, introducing a specific technique known as 4-7-8 breathing. |
| 4 | 1 | In-depth recap of the key learning from previous weeks, including the influence of mood on cognition, the formation of mental habits, the initiation of body scan practices, and the potency of breathing meditations. Following this, the focus shifts to the management of negative thoughts and emotions. |
|  | 2 | Scrutinises the relationship between negative events and their impact on self-perception, examining how we are affected by difficult situations. |
|  | 3 | Concentrates on the experiences of annoyance and discomfort, teaching how body scans can be employed to approach such sensations with kindness and acceptance instead of aversion. |
|  | 4 | Explores how breathing techniques can be used as a mechanism to interrupt automatic thinking and instil present-moment awareness, featuring a specific technique designed for this purpose. |
|  | 5 | Serves as a recap of week four's learning points, with an emphasis on applying the acquired knowledge and techniques in real-world contexts. |
| 5 | 1 | Revisits the body scan practice, which serves as an anchoring technique, and explores the concept of decoupling emotional thinking from reflection to achieve clearer understanding of events, facilitated through a guided meditation. |
|  | 2 | Continues the practice of body scans and focuses on the management of challenging emotions and feelings, specifically addressing internal self-talk and judgments. |
|  | 3 | Starts with a body scan and introduces a brief visualisation exercise aimed at altering perceptions of life's more challenging aspects. |
|  | 4 | Commences with the body scan and delves into the feelings of irritation and annoyance, exploring how mindfulness training can serve as an effective tool to mitigate these emotional responses. |
|  | 5 | Begins with a body scan, then recaps the week’s key learning around dealing with difficult situations and emotions, and is followed by a short breathing exercise. |
| 6 | 1 | Commences with a breathing exercise, emphasising its role as a core mindfulness practice, and then introduces the seminal concept that "thoughts are not facts," underscoring its pivotal role in cognitive restructuring. |
|  | 2 | Starts with a breathing exercise and then elaborates on the idea of "pausing in the moment," exploring the skill to momentarily disengage and gain perspective in various situations. |
|  | 3 | Begins with a gentle breath meditation and addresses the internal obstacles and resistances that can hinder mindfulness practice, supplemented by an exercise to help identify these barriers and potential resources to overcome them. |
|  | 4 | Opens with a breath meditation and investigates the correlation between emotions and somatic experiences. |
|  | 5 | Encapsulates the week's principal learnings and involves a thought-noticing exercise at the lake, following an initial breathing meditation. |
| 7 | 1 | Delves into understanding the inclination to be self-critical, concluding with an exercise designed to enhance self-image and self-esteem. |
|  | 2 | Investigates the interconnectedness of mood and activities, discussing how certain activities can elevate mood and the challenges that arise when motivation wanes. |
|  | 3 | Considers the role of intention and motivation in task completion, particularly when confronted with low mood. |
|  | 4 | Addresses the theme of suffering, elucidating how it is an inherent part of human experience that can be leveraged for personal growth. |
|  | 5 | Summarises the principal learnings and transitions into a stretch exercise. |
| 8 | 1 | Extensive review of each week, accompanied by a brief thought-noticing exercise. |
|  | 2 | Includes a longer body scan, aimed at deepening awareness of bodily sensations and emotional states. |
|  | 3 | Examines life challenges and explores how MBCT techniques can be employed to navigate complex situations and emotions. |
|  | 4 | Directs attention to specific life areas where people might like to apply mindfulness skills. |
|  | 5 | Features a brief meditation and concludes with a comprehensive summary, designed to reinforce key learnings and prepare participants for continued mindfulness practice in their daily lives. |

*Note:* MBCT = mindfulness-based cognitive therapy.
