# Supplementary material for: Virtual reality mindfulness-based cognitive therapy for reducing psychological distress in people with depression and/or anxiety symptoms: two uncontrolled feasibility studies
Source: Pilot Feasibility Stud. 2026 Aug 4;12:154. doi: 10.1186/s40814-026-01892-w (PMC13721488; doi:10.1186/s40814-026-01892-w)
Supplement: Supplementary file 4 [file 40814_2026_1892_MOESM4_ESM.docx]

Additional File 4. Data completeness for secondary outcome measures.

|  |  | **Pre** | | **Post** | |
| --- | --- | --- | --- | --- | --- |
|  | **Outcome measure** | **Missing** | **Completed**  **N (%)** | **Missing** | **Completed**  **N (%)** |
| Study 1 | PHQ-9 | 1 | 34 (97%) | 13 | 22 (63%) |
| (N = 35) | GAD-7 | 1 | 34 (97%) | 13 | 22 (63%) |
|  | WEMWBS | 2 | 33 (94%) | 15 | 20 (57%) |
|  | SMQ | 2 | 33 (94%) | 15 | 20 (57%) |
|  |  |  |  |  |  |
| Study 2 | PHQ-9 | 3 | 45 (94%) | 22 | 26 (54%) |
| (N = 48) | GAD-7 | 3 | 45 (94%) | 22 | 26 (54%) |
|  | WEMWBS | 8 | 40 (83%) | 25 | 23 (48%) |
|  | SMQ | 8 | 40 (83%) | 25 | 23 (48%) |

*Note:* GAD-7 = Generalized Anxiety Disorder-7. PHQ-9 = Patient Health Questionnaire-9. SMQ = Southampton Mindfulness Questionnaire. WEMWBS = Warwick-Edinburgh Mental Wellbeing Scale.
